# The Pathways study: a cohort study of new food-aid users in rural, semi urban, and urban areas of Quebec, Canada

**DOI:** 10.1186/s12889-023-16393-1

**Published:** 2023-08-24

**Authors:** Federico Roncarolo, Geneviève Mercille, Mylene Riva, Elsury Pérez, Rosanne Blanchet, Mabel Carabali, Marie-Pierre Sylvestre, Louise Potvin

**Affiliations:** 1 Centre de Recherche en Santé Publique (CReSP), CIUSSS du Centre-Sud-de-L’Ile-de-Montréal et Université de Montréal, Montréal, QC H3C 3J7 Canada; 2 Chaire de Recherche du Canada Approches Communautaires et Inégalités de Santé, Université de Montréal, Montréal, QC Canada; 3 Department of Nutrition, Faculté de Médecine, Université de Montréal, Montréal, QC Canada; 4 Department of Geography, McGill University, Canada Research Chair in Housing, Community and Health, Montréal, QC Canada; 5 Department of Social and Preventive Medicine, École de Santé Publique, Université de Montréal, Montréal, QC Canada; 6 Department of Epidemiology, Biostatistics, & Occupational Health, McGill University, Montréal, QC Canada; 7 Centre de Recherche du CHUM, Montréal, QC Canada

**Keywords:** Food insecurity, Food pantries, Food banks, Population research, Vulnerable populations, Cohort, Trajectories, Food assistance

## Abstract

**Background:**

While considerable research has been conducted on household food insecurity (HFI), little research has examined the effects of food donation programs on users’ living conditions. The Pathways study was established to investigate the long-term effects of food donation programs on food insecurity as well as other critical outcomes, such as diet, health, and social support. Herein, we describe the design of the Pathways Study and the participants’ characteristics at baseline.

**Methods:**

The Pathways study is a prospective cohort study of 1001 food-aid users in Quebec (Canada). We recruited newly registered users of food donation programs from 106 community-based food-aid organizations that partnered with the study. Baseline data were collected through face-to-face interviews from September 2018 to January 2020, with planned follow-up interviews at 12 and 24 months after enrollment. Household food insecurity, diet, food competencies, food shopping behaviors, perceived food environment, health status, social support and isolation, sociodemographic characteristics, housing conditions, negative life events, and the impacts of COVID-19 were assessed with validated questionnaires.

**Results:**

The cohort included 1001 participants living in rural (*n* = 181), semi-urban (*n* = 250), and urban areas (*n* = 570). Overall, household food insecurity was reported as severe among 46.2% and moderate in 36.9% of participants. Severe household food insecurity was more prevalent in rural (51.4%) and urban (47.8%) areas compared to semi-urban (39%) areas. Overall, 76.1% of participants reported an annual income below C$20,000. Half (52%) had low education levels (high school or lower), 22.0% lived in single-parent households, and 52.1% lived alone. Most (62.9%) experienced at least one major financial crisis in the preceding year.

**Conclusions:**

Results show that newly registered users of food donation programs often have low-income and severe food insecurity, with major differences across geographical locations. The Pathways study is the first study designed to follow, over a 2-year period, a cohort of newly registered users of food donation programs and to quantify their trajectories of service use. Findings from the Pathways study might help adapt the community response to the strategies used by food-insecure households to feed themselves.

## Background

Households experience food insecurity when they are unable to access a sufficient quantity of affordable and culturally appropriate, nutritious food due to financial constraints [1]. Household food insecurity (HFI) is a critical public health concern and its impacts on physical and mental health are well documented [2–5]. HFI is strongly associated with poverty [6, 7], and often occurs after life-changing events (e.g., loss of job, sickness, divorce, economic crisis, climate-related disaster) that destabilize households [8, 9]. In 2021, HFI affected 15.9% of households in Canada, representing 5.8 million individuals, including close to 1.4 million children under age 18 [1].

In Canada, as in most high-income countries, food donation programs, often implemented as local food banks offering food baskets for free or at a symbolic price, are the cornerstone of the societal response to HFI [10, 11]. Whether this represents an adequate solution to HFI is debated [10, 12–14]. Some argue that food donation might offer temporary relief and should be one component of a more comprehensive food security system [15]; in addition, it may act as a point of entry to a broader range of capacity-building programs to help households escape or mitigate the impact of poverty. Others contend that food donation programs should be abolished because they mask social injustices at the root of HFI, arguing for policies to be developed to eradicate the root causes of HFI, e.g., poverty [16]. This paper presents the Pathways study, a cohort of newly registered users of food donation programs, designed to assess the long-term impact of food donation programs on household food insecurity and other critical outcomes such as diet, health, and social support.

### Rationale of the Pathways study

Food donations alone are likely neither an adequate nor a sustainable response to the need for healthy and affordable food for households experiencing food insecurity [17–19]. Past studies have pointed out that many households experiencing food insecurity do not use food donation programs [10, 20]: feelings of stigmatization as well as poor quality and small quantities of food are often given as barriers to their use [18, 21–24]. Still, many community organizations complement food donations with capacity-building programs aimed at improving skills and empowering users. These programs include collective gardens, collective kitchens, food-buying groups, and budget counselling [25]. Many practitioners believe that combining food donations with capacity-building programs, as well as with a whole range of other community services (i.e., housing; education programs for adults; job programs, etc.) that increase the probability of transitioning out of poverty, represent a long-term solution to HFI [26]. In fact, studies have documented that capacity-building programs are associated with increases in the nutritional value of acquired food [25, 27], social integration, and solidarity [27–29], along with increases in the local availability of low-cost quality food [30, 31].

Yet, most studies assessing the impact of food donation programs have not considered the types, mandates, or contexts of the organizations that deliver them or the range of interventions they offer [19]. Furthermore, most of these studies are cross-sectional, and thus offer limited opportunity and validity to (1) estimate the effect of food donation programs on HFI; (2) understand how people interact with programs, i.e., whether programs are used as a one-time support in emergency cases or in a sustained or occasional way; and (3) assess whether and how these programs become a regular and/or important source of food acquisition once people start using them. To our knowledge, only a limited number of studies have considered new users compared to long-term users. In the U.S., Berner et al. distinguished between supplemental users and emergency users, who asked for food aid for the first time [32]. Kicinski defined new users as those who had turned to food banks for less than 2 years [33], whereas, in Canada, Black and Seto analyzed the use of food banks over time, distinguishing occasional from long-term users [17].

Since people living in poverty [34] exhibit major differences depending on whether they live in urban or rural areas, the geographical setting of food security studies is an important consideration. For instance, compared to poor urban working-class individuals, poor rural working-class individuals are more likely to be older, live with someone, and be part of a two-earner couple with children. Moreover, they are less likely to hold a university degree [34]. While Canadian data indicate that HFI is almost as prevalent in rural areas (11%) as in urban centers (13%) [35], most studies on HFI interventions are either conducted in urban and metropolitan settings [19] or do not disclose the location [23]. Thus, due to the unique local contexts and individual characteristics, including poverty distribution, HFI interventions differ across the urban–rural spectrum [36–38], and findings are not necessarily generalizable across settings.

Longitudinal studies that follow participants from when they first start using food donation programs are needed to describe the trajectories of the use of food donation programs (e.g., occasional resource or regular source of food acquisition) and to evaluate their effect on HFI and other related outcomes. Likewise, studies considering the geographical context of food banks and of their users, as well as the type of programs offered by community organizations in addition to food donation programs should be conducted. The Pathways study was designed to address these knowledge gaps.

The first of its kind, the Pathways study was designed to estimate the long-term impact of food donation programs on HFI and other outcomes such as diet, health, and social support among a cohort of newly registered users of food donation programs. It also aims to identify conditions that might facilitate the transition from food donation to capacity-building programs. The specific objectives of the Pathways study are to: (1) identify pathways for the use of food security programs and understand the drivers associated with most common pathways; (2) compare pathways of program use among new users in community organizations offering only food donation programs only (FD) and new users of food donation programs in community organizations that also offer food-related capacity-building programs (FD + CBP); and (3) quantify the relationships between pathways of donation program use and food security, diet, mental and physical health, and social integration. This paper presents the design of the Pathways study and the characteristics of participants at baseline, contrasting both the three different areas (rural semi-urban and urban) and the Montreal region versus the other three regions participating in the study.

### Methods

The Pathways study is conducted in the province of Québec, Canada. It was developed in close partnership with public health, provincial, and regional organizations (i.e., Food banks Quebec) that contributed to its design and implementation. The Pathways study is funded by the Canadian Institutes of Health Research (grant# omitted for blind review), with additional financial support from the Quebec Ministry of Health and Social Services, the Foundation of Greater Montreal, and Mission Inclusion. We received ethical approval from the Research Ethics Board of the Université de Montréal (n. certificate blinded for peer review).

### Design, setting, and target population

Community organizations (through which users were recruited) were situated in rural, semi-urban, and urban areas in four administrative regions in the province: the Island of Montreal, Lanaudière, Mauricie-Centre-du-Québec, and Estrie. All the regions have urban, semi-urban, and rural settings except for Montreal, which is solely urban and semi-urban.

The study population consisted of individuals who used a food donation program offered by a community organization in one of the study regions for the first time in the preceding 12 months. Over the course of the Pathways study, participants were interviewed three times: at baseline (T_0_), defined as within six months after their first use of a food donation program (September 2018–January 2020); at 12 months follow-up (T_1_; September 2019–February 2021); and at 24 months follow-up (T_2_; September 2020–February 2022).

### Recruitment

The sample of participants was assembled in two phases. We first recruited community organizations in the four regions from which we then recruited first-time users.

#### Recruitment of community organizations

Across the four regions, we inventoried a total of 423 community organizations offering FD or FD + CBP. All organizations were contacted to assess eligibility, explain the study, and invite them to help the research team recruit participants. Organizations offering intermittent food donations (e.g. at Christmas) and those providing meals to specific groups (e.g., Breakfast Club) were excluded. Overall, 149 organizations agreed to participate, 17 organizations could not be reached, 160 did not meet inclusion criteria, and 97 refused to participate. We conducted a 30-min phone interview with one key informant from each organization (usually the director), asking them to describe the activities and services offered by their organization (i.e., whether they provided FD or FD + CBP programs) and to confirm their willingness to help identify and recruit first-time users of their food donation program. Of the 149 organizations, 117 provided the names of potential participants*.* We were unable to enroll participants from 11 of these organizations. Ultimately, participants were recruited from 106 organizations (71.1% of organizations that agreed to participate). The recruited organizations were categorized by type of program(s) offered: FD (43 organizations: 40.6%) or FD + CBP (63 organizations: 59.4%) and by their setting: urban (48 organizations: 45.3%); semi-urban (29 organizations: 27.4%), and rural (29 organizations: 27.4%). More details on recruitment are provided elsewhere [citation omitted for peer review].

#### Recruitment of participants and retention

Based on a previous study of new food banks users in the region of Montreal [39], we estimated that, with 1,008 participants at T_2_, *p* < 0.05, power 0.80, and intra-class correlation coefficients of 0.05 for HFI, 0.006 for self-rated physical health, 0.01 for self-rated mental health, and 0.106 for social support, we could detect small effect sizes. Based on our retention strategy and previous study results [39], we expected 70% retention at T_1_ and 80% at T_2_. With these retention rates, we estimated that 1800 respondents would be needed at T_0_.

Contact details (names, telephone numbers, or emails) for a total of 1784 individuals willing to participate in the Pathways study were provided by participating food donation organizations or were obtained directly by interviewers on site during food-aid distribution. In total, 1001 participants were enrolled at baseline (56.1%). Of the 783 participants who were not interviewed, 227 (29%) could not be contacted by the research team; 47 (6%) did not meet our inclusion criteria (see Table 1); 125 (16%) were not interested or did not have time to participate; 235 (30%) did not participate for other reasons; and 149 (19%) did not show up to the face-to-face baseline interview appointment. Of the 1001 people enrolled in the study, 181 (18.1%) were recruited in rural settings, 250 (25%) in semi-urban settings, and 572 (57%) in urban settings (Table 2). Considering the type of organizations, 379 (37.9%) enrolled participants were recruited from FD organizations and 622 (62.2%) from FD + CBP organizations.

**Table 1:** Recruitment of participants: exclusion criteria

Inclusion criteria	Exclusion criteria	Justification
Adults aged 18 years and over who registered and used a food donation program for the first time in the past 6 months in one of the 117 participating community organizations who provided participants names and coordinates	Individual participating in a food donation program in another organization in the preceding 12 months	
	Age over 63 years at baseline	Individuals over this age are eligible for the senior citizens guaranteed income supplements over the course of the study (which would alleviate a primary cause of food insecurity)
	Individuals in situation of homelessness	Homeless people constitute a small minority of users of the organizations of interest in this study We were not able to assure adequate follow-up of participants with no home address
	Individuals living with a person already enrolled in the study	
	Individuals who do not speak French nor English	The diversity of languages spoken in Montreal is such that translating/back-translating questionnaires or using interpreters would have incurred significant costs and introduced measurement bias. In addition, new immigrants to Quebec are enrolled in French integration classes free-of-charge and most quickly become functional in French

**Table 2:** Sample size and retention rate by region and setting^a^

	**Total**	**Estrie**	**Mauricie Centre du Québec**	**Lanaudière**	**Montreal**
	**n %**	**n %**	**n %**	**n %**	**n %**
**Rural**					
T_0_	181	74	70	37	n.a
T_1_ retention	138 (76.2%)	52 (70.3%)	56 (80.0%)	30 (81.1%)	n.a
T_2_ retention	120 (66.3%)	44 (59.5%)	51 (72.9%)	25 (67.6%)	n.a
**Semi-urban**					
T_0_	250	52	60	39	99
T_1_ retention	199 (79.6%)	39 (75.0%)	49 (81.7%)	28 (71.8%)	83 (83.8%)
T_2_ retention	177 (70.8%)	33%	46 (63.5%)	23 (59%)	75 (75.8%)
**Urban**					
T_0_	570	155	131	49	235
T_1_% retention	408 (71.6%)	92 (59.4%)	98 (74.8%)	31 (63.3%)	187 (79.6%)
T_2_% retention	345 (60.5%)	70 (45.1%)	85 (64.9%)	20 (40.8%)	170 (72.3%)
**Total**					
T_0_	1001	281	261	125	334
T_1_% retention	745 (74.4)%	183 (65.1%)	203 (77.8%)	89 (71.2%)	270 (80.8%)
T_2_% retention	642 (64.1%)	147 (52.3%)	182(69.7%)	68 (54.4%)	245 (73.4%)

^a^T_2_ retention rates are calculated on the T_0_ respondents

Of the 1001 participants at baseline (T_0_), 745 (74.4%) completed the 1-year follow-up interview (T_1_) and 642 (86.2%) completed the T_2_ survey (Table 2). The overall two-year retention rate of the study is 64.1%. The retention rate at T_2_ was slightly lower in urban areas compared to semi-urban and rural areas. Moreover, there are regional differences; the Montreal region had higher retention rates than the other three regions (Table 2).

### Data collection

At baseline, participants were interviewed face-to-face by trained interviewers. The 60-min bilingual (French/English) questionnaire had been mostly developed, validated, and used in a past study [39]. A similar questionnaire was administered at the two follow-ups, after one (T_1_) and 2 years (T_2_) respectively. Questions were added at T_1_ and T_2_ to assess in greater detail pathways of use of food security programs. At the mid-point of the first follow-up data-collection period (precisely on March 13 2020), the first lockdown related to the COVID-19 outbreak was implemented province-wide. Thus, for T_1_, only 50% of participants were interviewed face-to-face; the remaining half were interviewed remotely (over the phone or on web-based software). All T_2_ questionnaires were administered remotely. In addition, a section with questions assessing the impact of the COVID-19 pandemic was added to the T_2_ questionnaire.

#### Measures

*Stratification variablesFood security organization type at recruitment* was categorized in one of two types: (1) Food donation only (FD), which included organizations providing food baskets to users free-of-charge or at a symbolic price; and (2) Food donation plus capacity building program (FD + CBP), which included those organizations providing food donations as well as food-related capacity-building programs aimed at empowering users and fostering their social integration, while facilitating acquisition of quality food at lower cost (e.g., collective kitchens, collective gardens, food-buying groups, cooking or nutrition workshops).

*Geographical setting* comprises urban, semi-urban, and rural areas, defined according to the definitions of Statistics Canada’s Census Metropolitan Areas (CMAs) and Census Agglomerations (CAs) [40] in conjunction with Regional County Municipalities’ development plans used by administrative regions in Quebec to structure development and to allocate resources to municipalities. Urban settings were defined as urban centers with a core population ≥ 50,000 for CMAs and ≥ 10,000 for CAs. They are characterized by higher population and building density as well as more mixed land uses (residential, commercial, services, industries). Considering CMAs and CAs, semi-urban settings are generally located on the periphery of urban centers; they have relatively dense built environments and mixed land uses. Outside of CMAs and CAs, semi-urban settings include municipalities with ≥ 5000 inhabitants with an important land-use mix, characterized by low diversity; are often close to rural areas; and are not contiguous to urban centers. Rural settings are characterized by low population density and a lack of diversity in land use. They are composed of municipalities inside or outside CMAs and CAs. There are no rural areas within the Montreal CMA.

#### Primary and secondary outcome variables

Pathways of use of food donation programs and household food security are the main outcome variables in the Pathways study. Other variables of interest investigated in the study are diet, food-related competencies, food acquisition patterns, physical and mental health, social support and isolation,

*Pathways of use of food donation program* was assessed at T_1_ and T_2_ by asking participants about their use of food donation programs in each month of the preceding year with the following questions: “Since our last interview on [DATE] have you used food donation programs? If yes, did you use it in [list all months]?”. The use of capacity-building programs was also investigated with similar questions.

*Household food security* was measured using the Household Food Security Survey Module (HFSSM), a well-validated, 18-item scale that measures inadequate or insecure access to food due to financial constraints [41]. Developed by the U.S. Department of Agriculture (USDA) [42], it was subsequently approved by Health Canada as the measurement tool for HFI in Canada [43]. The questions differentiate the experiences of adults from those of children, recognizing that in households with children, adults might compromise their own food intake to reallocate scarce resources for children. Based on their responses to the HFSSM, households were categorized into one of the four food-security categories: food secure (households had access to enough food at all times throughout the previous year), marginally food secure (some concern or problem of food access), moderately food insecure (compromises in the quality and/or quantity of food consumed), and severely food insecure (extensive compromises, including reduced food intake) [1, 43].

*Diet* was measured from an adapted version of the Short Diet Questionnaire (SDQ), developed and validated for use in Quebec [44]. Four items were dropped from the SDQ for the purpose of the study, leaving a 32-item food-frequency questionnaire (FFQ), used to assess usual consumption over the previous 12 months. The frequency of restaurant meals and meal frequency were also evaluated through ad hoc multiple-choice questions.

*Food-related competencies* were assessed with eight questions related to food planning and preparation based on questions from the Canadian Community Health Survey. Examples of questions are “Do you try to avoid foods that are high in sugar, salt, and fat?” or “In the past 12 months, did you shop with a grocery list?” [45].

*Self-rated physical and mental health* was assessed with the SF-12v2 [46], which generates two component summary scores, the Physical Component Summary (PCS) and the Mental Component Summary (MCS). We further assessed psychological distress with the Kessler six-item scale [47]. A score of 13 or higher on the Kessler six-item scale indicates psychological distress.

*Perceived social support* was measured with a modified version of the Multidimensional Scale of Perceived Social Support (MSPSS) [48], which consists of 12 items evaluating perceived social support from friends, family, and a special other.

*Social isolation* was measured with five questions from the Social Isolation subscale of the Nottingham Health Profile scale [49].

*Food acquisition indicators.* The type of food store used, its location, frequency of food purchasing, frequency of use of the food donation program, transportation mode and travel time to the most commonly used grocery store were investigated. Summertime fruit and vegetables market use and gardening, either at home or in a community garden, were also assessed.

#### Covariates

Perceived food environment, negative life events, socio-demographic characteristics and housing conditions are covariates considered in this research. The impact of the COVID-19 pandemic and lockdown was investigated through specific questions during the second follow-up (T_2_)_._ Whenever possible, we used validated measures associated with food insecurity and/or food aid attendance. These associations were described in the scientific literature or highlighted as potentially relevant by the study’s knowledge user partners.

*Perceived food environment* was evaluated with the Perceived Food Environment Questionnaire, which assesses the quality, variety, quantity, and affordability of healthy food in the reference grocery store, as well as the accessibility of restaurants and of a wide variety of food near the home [50]. Difficulties in shopping (physical and financial difficulties) were also assessed with questions previously tested [51].

*Socio-demographic characteristics (i.e. employment, income, source of income) and housing conditions (i.e., dwelling type and characteristics)* of participants were assessed with questions from the Canadian Community Health Survey and the Canadian census [45].

*Negative life events* in the year preceding the survey were used to evaluate events that might have brought people to ask for food aid; these were measured with questions from the Holmes and Rahe stress scale [52].

*Impact of the COVID-19 pandemic* was assessed with questions added to the T_2_ questionnaire to document changes in the household’s financial situation, changes in employment (job losses, job changes, or temporary layoffs), and to determine if the respondent or any close relative had been affected by COVID-19.

### Analysis

Chi square tests were performed to assess differences between proportions. For continuous variables(age, number of people in the household, food donation monthly frequency, months of food-aid participation before recruitment, SF12 v2 scale scores, and number of events, Student t-tests were performed to assess differences between the Montreal area and the other regions, and ANOVA to assess differences between settings (urban, semi-urban, rural).

## Results

Table 3 presents the descriptive statistics of participants at baseline. On average, participants responded to our first questionnaire 2.1 months after having registered in a food donation program for the first time. Overall, participants in the Pathways study lived in food-insecure households and had several vulnerabilities. Close to half (46.2%) the participants lived in severe food-insecure households in the preceding year, and 36.9% lived in a moderate food-insecure household. The mean age was 40.4 years; 60.9% of participants identified as women. Seventy-six percent reported an annual household income under C$20,000; 51.1% reported having completed high school or less. Over 20% were experiencing psychological distress. Most participants were renters (89.8%) and lived alone (52.1%). About 20% reported living in dwellings requiring major repairs.

**Table 3:** Descriptive characteristics of first-time users of food aid at baseline for the total sample by geographic setting and region^a^

	**Total** **n (%)**	**Rural** **n (%)**	**Semi-urban** **n (%)**	**Urban** **n (%)**	***p*-value**^**b**^	**Montreal** **n (%)**	**Other regions n (%)**	***p*-value**^**c**^
	1001 (100%)	181 (100%)	250 (100%)	570 (100%)		334 (100%)	667 (100%)	
**Socioeconomic Characteristics**								
*Gender*					0.002			0.354
Male	389 (38.9)	53 (29.3)	82 (32.8)	254 (44.6)		136 (40.7)	253 (37.9)	
Female	610 (60.9)	128 (70.7)	168 (67.2)	314 (55.1)		197 (59.0)	413 (61.9)	
Other	2 (0.2)	–	–	2 (0.4)		1 (0.3)	1 (0.1)	
*Age*, mean (SD)	40.4 (11.9)	40.4 (12.5)	41.8 (11.6)	39.8 (11.8)	0.11	40.8 (10.9)	40.2 (12.4)	0.47
*Country of birth*					< 0.001			< 0.001
Canada	774 (77.4)	173 (95.6)	181 (72.4)	420 (73.8)		158 (47.3)	616 (92.5)	
Elsewhere	226 (22.6)	8 (4.4)	69 (27.6)	149 (26.2)		176 (52.7)	50 (7.5)	
*Household income*					0.066			0.100
< $20,000	737 (76.1)	124 (69.3)	180 (74.1)	433 (79.2)		245 (78.3)	492 (75.0)	
$20,000–$29,999	109 (11.2)	29 (16.2)	27 (11.1)	53 (9.7)		24 (7.7)	85 (13.0)	
$30,000–$39,999	59 (6.1)	12 (6.7)	21 (8.6)	26 (4.8)		21 (6.7)	38 (5.8)	
≥ $40,000	64 (6.6)	14 (7.8)	15 (6.2)	35 (6.4)		23 (7.3)	41 (6.3)	
*Highest education level in household*					< 0.001			< 0.001
High school or lower education	512 (51.5)	118 (65.3)	127 (51.0)	267 (47.3)		107 (32.4)	405 (60.9)	
Professional program	148 (14.9)	29 (16.0)	32 (12.9)	87 (15.4)		36 (10.9)	112 (16.8)	
CEGEP	129 (13.0)	22 (12.2)	37 (14.9)	70 (12.4)		44 (13.3)	85 (12.8)	
University	205 (20.6)	12 (6.6)	53 (21.3)	140 (24.8)		142 (43.0)	63 (9.5)	
Other	1 (0.1)	–	–	1 (0.2)		1 (0.3)	–	
*Employment status*					< 0.001			< 0.001
Working	159 (15.9)	27 (14.9)	43 (17.2)	89 (15.9)		59 (17.8)	100 (15.1)	
Actively looking for work	187 (18.7)	23 (12.7)	50 (20.0)	114 (20.0)		63 (19.0)	124 (18.7)	
Studying	113 (11.3)	9 (5.0)	29 (11.6)	75 (13.2)		62 (18.7)	51 (7.7)	
At home	143 (14.3)	49 (27.1)	28 (11.2)	66 (11.6)		34 (10.2)	109 (16.4)	
Long-term sick leave	150 (15.0)	24 (13.3)	47 (18.8)	79 (13.9)		48 (14.5)	102 (15.4)	
Inactive	108 (10.8)	21 (13.3)	22 (8.8)	65 (11.4)		20 (6.0)	88 (13.3)	
Other	136 (13.6)	27 (14.9)	30 (12.0)	79 (13.9)		46 (13.9)	90 (13.6)	
**Household Composition and Housing Conditions**								
*Household composition*					0.002			< 0.001
Single-parent	220 (22.0)	51 (28.2)	68 (27.2)	101 (17.7)		71 (22.2)	146 (21.9)	
Couple with children	182 (18.2)	35 (19.3)	54 (21.6)	93 (16.3)		89 (26.6)	93 (13.9)	
Couple without children	67 (6.7)	14 (7.7)	14 (5.6)	39 (6.8)		18 (5.4)	49 (7.3)	
Unattached, living alone or with others	522 (52.1)	80 (44.2)	111 (44.4)	331 (58.1)		148 (44.3)	374 (56.1)	
Other	10 (0.1)	1 (0.6)	3 (1.2)	6 (1.1)		5 (1.5)	5 (0.7)	
*Number of people in the household* Mean (SD)	2.3 (1.4)	2.5 (1.6)	2.2 (1.3)	2.3 (1.3)	0.146	2.4 (1.3)	2.2 (1.4)	0.033
*Dwelling ownership*					< 0.001			< 0.001
Yes	101 (10.1)	32 (17.8)	27 (10.8)	42 (7.4)		17 (5.1)	84 (12.6)	
No	898 (89.8)	148 (82.2)	223 (89.2)	527 (92.6)		317 (94.9)	581 (87.4)	
**Food Security and Program Use**								
*Household food-security status*					0.032			< 0.001
Food secure	84 (8.4)	10 (5.5)	18 (7.2)	56 (9.8)		47 (14.1)	37 (5.5)	
Marginally food insecure	84 (8.4)	16 (8.8)	30 (12.0)	38 (6.7)		30 (9.0)	54 (8,1)	
Moderately insecure	369 (36.9)	62 (34.3)	104 (41.8)	203 (35.7)		147 (44.3)	222 (33.3)	
Severely insecure	462 (46.2)	93 (51.4)	97 (39.0)	272 (47.8)		108 (32.5)	354 (53.1)	
*Food donation is the first access to organizations*					0.053			< 0.001
Yes	856 (85.6)	145 (80.1)	213 (85.2)	498 (87.4)		302 (90.4)	554 (83.1)	
No	145 (14.5)	36 (19.9)	37 (14.8)	72 (12.6)		32 (9.6)	113 (16.9)	
*Food donation monthly frequency, *Mean (SD)	2.4 (1.4)	2.1 (1.3)	2.5 (1.3)	2.4 (1.4)	0.001	2.6 (1.4)	2.1 (1.4)	< 0.001
*Months of food-aid participation before recruitment *Mean (SD)	2.1 (1.7)	2.2 (1.6)	2.5 (1.8)	2 (1.7)	< 0.001	2.5 (1.8)	2 (1.7)	< 0.001
*Past use of collective kitchens, collective gardens, or buying groups*					0.167			0.731
Yes	174 (17.4)	36 (19.9)	34 (13.6)	104 (18.2)		60 (18)	114 (17.1)	
No	827 (82.6)	145 (81.1)	216 (86.4)	466 (81.8)		274 (82)	553 (82.9)	
**Health**								
*SF12 v2 scale*								
Physical Component Summary, Mean (SD)	45.6 (13.3)	45.4 (13.3)	44.7 (13.1)	46.1 (13.3)	0.353	46.4 (12.6)	45.3 (13.6)	0.217
Mental Component Summary, Mean (SD)	40.7 (12.7)	40.8 (12.5)	40.3 (13.4)	40.8 (12.5)	0.873	43.0 (11.2)	39.6 (13.3)	< 0.001
*Kessler score*					0.126			0.102
Lower than 13	758 (77.2)	141 (78.3)	177 (72.2)	440 (79.0)		500 (75.8)	258 (80.1)	
13 or higher	224 (22.8)	39 (21.7)	68 (27.6)	117 (21.0)		160 (24.2)	64 (19.9)	

*SD* Standard deviation

^a^Missing values are not shown

^b^ Estimated using chi square tests excepted for age, number of people in the household, food donation monthly frequency, months of food-aid participation before recruitment and SF12 v2 scale scores that were estimated using Student t-tests

^c^ Estimated using chi square tests excepted for age, number of people in the household, food donation monthly frequency, months of food-aid participation before recruitment and SF12 v2 scale scores that were estimated using ANOVA

There were statistically significant differences between participants in rural, semi-urban and urban areas, both in terms of their demographic profiles and socioeconomic characteristics. While women represent 70.7% of participants in rural areas, they represent a little more than half (55.1%) in urban areas. With regards to employment status, 12.7% of respondents were looking for a job in rural areas compared to 20% in semi-urban and urban areas. The level of education attained was higher among participants in urban and semi-urban areas compared to rural areas; household income was lower in urban areas than in semi-urban and rural areas, despite the fact that the average number of people in households was not different. The proportion of participants who lived in severe food-insecure households in the previous year was 51.4% in rural areas, 47.8% in urban areas, and 39.0% in semi-urban areas. No differences were found concerning scores for mental- and physical-health scales across living areas. Almost 60% of participants in urban areas lived alone; this proportion was significantly lower in rural and semi-urban areas. More participants in rural areas were homeowners and reported their homes were in need of major repairs compared to participants in urban areas.

Comparing Montreal with all other areas, we found that 52.7% of participants in Montreal reported being born outside of Canada; this proportion was 7.5% in the other regions. There were more students in Montreal, and the education level of participants was higher; fewer people lived alone there compared to the other regions. Fewer participants in Montreal lived in households that experienced moderate or severe food insecurity (76.8%) compared to the other regions (86.4%). Moreover, they accessed food aid more frequently: 2.6 times per month in Montreal compared to 2.1 times per month in the other regions.

As for stressful life events in the year prior to the survey, respondents reported an average of 3.1 events (Table 4). Almost 40% of respondents reported a serious illness for themselves and 28.4% for a close relative. Sixty-three percent reported a major financial crisis with a high of 72.4% in rural areas. The number of respondents who reported becoming unemployed was lower in rural areas (23.2%) compared to urban areas (33.5%). One in four respondents (24.2%) experienced a separation or divorce. Only 76 (7.6%) of participants reported no stressful life events in the year prior to first food bank use.

**Table 4:** Stressful events experienced in the past year for the total sample and by geographic setting^a^

	**Total** **n (%)**	**Rural** **n (%)**	**Semi-urban** **n (%)**	**Urban** **n (%)**	***p*-value**^**b**^	**Montreal** **n (%)**	**Other regions** **n (%)**	***p*-value**^**c**^
	1001 (100%)	181 (100%)	250 (100%)	570 (100%)		334 (100%)	667 (100%)	
Serious illness, injury, or assault of the person	370 (37)	64 (35.4)	94 (37.8)	212 (37.3)	0.867	127 (37.0)	243 (36.5)	0.610
Serious illness, injury, or assault of a close relative	283 (28.4)	49 (27.1)	75 (30)	159 (28)	0.776	99 (29.8)	184 (27.6)	0.469
Death of a parent, child, or spouse	106 (10.6)	24 (13.3)	26 (10.4)	56 (9.9)	0.434	34 (10.2)	72 (10.8)	0.766
Death of a close family friend or another relative	346 (34.8)	63 (35.2)	92 (36.8)	191 (33.8)	0.705	118 (35.5)	228 (34.4)	0.731
Separation due to marital difficulties	242 (24.2)	46 (25.4)	57 (22.9)	139 (24.4)	0.823	62 (18.6)	180 (27)	0.003
Serious problem with a close friend, neighbor, or relative	330 (33.0)	56 (30.9)	76 (30.5)	198 (34.7)	0.403	88 (26.4)	242 (36.3)	0.002
Became unemployed	293 (29.4)	42 (23.2)	61 (24.4)	190 (33.5)	0.004	107 (32.3)	186 (27.9)	0.147
Looked for work unsuccessfully for more than 1 month	317 (31.7)	49 (27.1)	64 (25.6)	204 (35.8)	0.005	114 (34.1)	203 (30.4)	0.236
Major financial crisis	627 (62.9)	131 (72.4)	160 (64.5)	336 (59.2)	0.005	152 (45.9)	475 (71.3)	< 0.001
Problems with the police or court appearance	180 (18)	41 (22.7)	34 (13.6)	105 (18.6)	0.049	33 (9.9)	147 (22.1)	< 0.001
Something valued as lost or stolen	208 (20.9)	36 (19.9)	52 (21.0)	120 (21.2)	0.934	55 (16.7)	153 (22.9)	0.023
Other (i.e., pregnancy, immigration, depression, suicide attempts)	107 (11.2)	19 (11.2)	33 (13.5)	55 (10.1)	0.367	31 (9.4)	76 (12.1)	0.201
Number of events, Mean (SD)	3.4 (2.1)	3.4 (1.9)	3.3 (2.0)	3.4 (2.2)	0.625	3.1 (2.2)	3.6 (2.0)	< 0.001

*SD* Standard deviation

^a^ Missing values are not shown

^b^ Estimated using chi square tests excepted for number of events that was estimated using Student t-tests

^c^ Estimated using chi square tests excepted for number of events that was estimated using ANOVA

The average number of stressful life events reported by participants was lower in Montreal (3.1) than the other regions (3.6). In addition, 45.9% of respondents in Montreal reported a major financial crisis in the preceding year compared to 71.3% of respondents in the other regions.

## Discussion

For the Pathways study, we assembled and followed up a cohort of newly registered users of food donation programs living in rural, semi-urban, and urban areas [53]. When compared to the Quebec 2016 population census data [54], our cohort was composed of people living in conditions of extreme vulnerability. Seventy-six percent had a household income below C$20,000 compared to 11.4% in the provincial population. About 23% of people living in Quebec aged 25 to 64 years reported high school as their highest education level compared to 52% among Pathways participants. These extreme conditions of vulnerability of people in our cohort were to be expected. Loh et al. [55] reported poorer health and worse mental-health conditions among food-bank users compared to the general population in England. In line with our study, Bhattarai et al. reported higher proportions of single-parent families and people living alone among food-bank users [56]. Lastly, in a UK cross-sectional study, Prayogo et al. reported that food-bank users experienced more financial strain, adverse life events, and food insecurity than other disadvantaged groups [57]. Nevertheless, none of these studies focused on new food-banks users or differentiated among geographical settings.

Studies have described how people often asked for food aid when their lives were majorly disrupted by stressful events, especially when the events impacted the household’s economic balance [32, 57, 58]. Herein, most respondents reported injuries or illnesses, death of a close relative, job loss, and marital separation in the year before accessing food aid. These events could have profoundly disrupted their day-to-day life and finances, leading them to ask for food aid. Despite people trying to overcome financial crisis by cutting down on food [32, 59], sometimes they had to resort to a last-resource strategy such as getting food from food banks [20]. The longitudinal results of the Pathways study will provide the means to assess the extent to which using food aid is just a temporary measure or if it becomes a service required for a long time and under what conditions.

The Pathways study will also provide new insights in exploring the differences among newly registered users of food donation programs living in rural, semi-urban, and urban areas, as well as in the Montreal metropolitan area compared to other administrative regions in the province. The literature on rural and urban poverty [34, 60] as well as the Pathways study underscore the different conditions that characterize vulnerability in urban and rural households. Consequently, the stressful life events that lead participants to prevail themselves of food donation might differ geographically, as we observed in the Pathways study. In addition, more urban participants in the Pathways study reported becoming unemployed in the preceding year compared to participants in semi-urban and rural areas; fewer reported having experienced a major financial crisis. The different experiences in life stressful events can then lead people to approach food banks differently in terms of attendance across geographical settings.

Participants who accessed food aid in Montreal seemed to have better conditions than those in the other regions. They were more likely to live in food-secure households. They also reported better health and fewer stressful life events in the preceding year than participants living in the other areas. It is possible that these participants were able to access food aid when their condition was less compromised because of a higher density of community organizations in Montreal [38]. Thus, they might have been able to access food aid earlier than participants in the other regions. It is also possible that the social stigma around accessing food aid is more acute in smaller towns or rural areas than in urban areas, which could influence access to food donation. Another possible explanation relates to the higher percentage of recent immigrants (who immigrated in the last 12 months) in Montreal (12%) compared to other regions (1%). Depending on their country of origin, they might score lower on the HFSSM due to conditions in their home countries (for instance, if they experienced prolonged famine) between Montreal and the other administrative regions. However, this information was not collected.

Most of the respondents in the Montreal region were not born in Canada, more than what we would expect from Montreal demographics (34.3%) [61]. Immigrants, regardless of gender, level of education, family type, or province of residence, were more likely to have low incomes compared to the Canadian-born population [62]. This might explain why, despite a higher level of education, households in Montreal did not have a higher income.

### Limitations

The Pathways study will have to address some limitations. First, despite having contacted all community organizations providing food aid in the selected regions, not all of them participated in the study. Moreover, due to difficulties in recruitment, we were unable to recruit the same number of people from FD and FD + CBP organizations [Reference omitted for peer review]. Second, our sample only included participants who were able to respond to the questionnaire in English or French. This criterion might have excluded some recent immigrants who did not understand the official languages and who often have worse social and economic outcomes [63]. Third, for reasons of feasibility, to avoid putting pressure on people to participate in the study, and in agreement with community organizations, we agreed that 6 months of participation in food-aid programs was an acceptable compromise in recruiting new food-aid applicants. Nevertheless, the potential effects of receiving food aid on food security and other dependent variables could have been already at play before doing the interview. Lastly, the pathways of use of food donation programs were assessed by asking participants about their use of food donation programs in each month of the preceding year. Recall bias cannot be excluded because some participants had a difficult time precisely remembering the months of attendance in food programs.

## Conclusion

To our knowledge, the Pathways study is the first study designed to follow a cohort of newly registered users of food donation programs to study their trajectories of service use over a 2-year period. In addition, it is the first study that contrasts first-time users in rural, semi-urban, and urban areas. Furthermore, this study focused on the impact of these trajectories on HFI status and on other critical outcomes such as diet, health, and social support of participants. All these characteristics make this study unique. The baseline analyses show that newly registered users of food donation programs face multiple vulnerabilities, such as low-income, precarious health conditions, and severe food insecurity, at a much higher level than the general population. The differences between geographical settings show that food aid users should not be considered monolithic: differences exist between those who use food aid in rural, semi-urban, and urban areas. These differences might affect access to services and the different needs of food-aid participants. Accordingly, they should be considered when planning, organizing, and providing food-aid strategies The findings of the Pathways study will provide a solid evidence base on the effects of food donation programs and will enable adaptation of the community response to the strategies that households use to feed themselves.

## Data Availability

The datasets generated and analyzed during the current study are not publicly available due to confidentiality agreements with community organizations and participants. They are available from the corresponding author in response to reasonable requests.
